# Improved efficiency of daratumumab treatment of multiple myeloma adopting the subcutaneous route: A micro‐costing analysis in three Italian hematology centers

**DOI:** 10.1002/cam4.6699

**Published:** 2023-11-09

**Authors:** Lorenzo Pradelli, Massimo Massaia, Elisabetta Todisco, Filippo Gherlinzoni, Anna Furlan, Maria La Targia, Elisabetta Grande, Ignazio Ezio Tripoli, Francesca Occhipinti, Francesco Comello, Fabrizio Iannello, Stefania Bellucci

**Affiliations:** ^1^ AdRes HE&OR Torino Italy; ^2^ S.C. Ematologia A.O. Santa Croce e Carle Cuneo Italy; ^3^ U.O. Ematologia ASST Valle Olona Busto Arsizio Busto Arsizio Italy; ^4^ Divisione di Ematologia Ospedale Cà Foncello di Treviso–ASL 2 Treviso Italy; ^5^ Janssen‐Cilag SpA Cologno Monzese Italy

**Keywords:** cancer management, clinical management, clinical observations, drug discovery and delivery, multiple myeloma

## Abstract

**Background:**

Daratumumab is a humanized monoclonal antibody approved for the treatment of adult patients with newly diagnosed or relapsed/refractory multiple myeloma (RRMM). Subcutaneous (SC) formulation proved to be non‐inferior in comparison with intravenous (IV) administration route. This study aimed at assessing the economic and time impact associated with the use of SC versus IV daratumumab in patients with RRMM from the perspective of the hematology center.

**Methods:**

This was a 5‐month multicenter time‐and‐motion cross‐sectional micro‐costing study conducted in three Italian hematology centers among adult patients diagnosed with RRMM with ongoing treatment with IV or SC daratumumab. Measurements were performed by an ad hoc App.

**Results:**

Nineteen (20%) IV and 76 (80%) SC administration procedures were measured. Patients spent a mean of 4.85 ± 0.91 or 1.08 ± 0.56 h in the hematology center to receive IV or SC daratumumab, respectively. Healthcare professionals (HCPs) spent a mean of 49.38 ± 16.13 and 20.37 ± 7.88 min of active working time to manage IV and SC administrations, respectively. The infusion chair was occupied for a mean of 4.85 ± 0.91 and 0.99 ± 0.55 h during IV or SC administration, respectively. On average, considering the costs due to HCP and chair time, materials, and overhead costs, every IV and SC administration costed €80.33 and 34.90, respectively.

**Conclusions:**

In conclusion, as compared with IV administration, SC daratumumab was associated with 78%, 59%, 80% savings in terms of patient time, HCP active working time, and infusion chair, respectively, and 56.6% budget savings.

## Graphical abstract

This study aimed at assessing the economic impact associated with the use of SC versus IV daratumumab in patients with RRMM from the perspective of the hematology center. We found that, as compared with IV administration, SC daratumumab was associated with 78%, 59%, 80% savings in terms of patient time, HCP active working time, and infusion chair, respectively, and 56.6% budget savings.

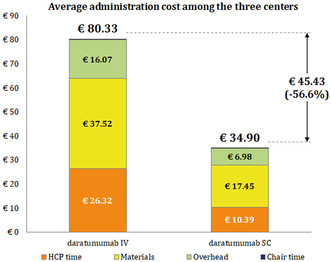

## 1 INTRODUCTION

Multiple myeloma (MM) is a blood cancer characterized by abnormal proliferation of a clone of plasma cells, which accumulate in bone marrow, all producing identical immune globulins (monoclonal component). Clinically, it is characterized by calcium elevation, renal insufficiency, anemia, and bone lytic lesions, usually indicated by the acronym CRAB.^1^ Its pathogenesis and underlying biological pathways have only been partially elucidated and are still under investigation.^1^

In Italy, MM accounts for 1.3% and 1.2% of all tumors in men and women, respectively, with incidence rates of 9.5 and 8.1 yearly new cases of MM every 100,000 male and female inhabitants. At national level, this means 2315 and 2098 new MMs in men and women, respectively. The incidence rates are notably stable in time and across regions. MM is an illness of the elderly, as the median age at diagnosis is 68 years: about 2% of diagnoses pertain patients under 40 years, while more than 60% concern individuals over 70 years.^1^

Daratumumab is a humanized monoclonal antibody directed against CD38 antigen. It is approved for the treatment of adult patients with newly diagnosed or relapsed/refractory multiple myeloma (RRMM), both as monotherapy and in combination with other antineoplastic agents.^2^

Daratumumab combinations are administered in cycles and treatment is usually given until progression or inacceptable toxicity. It was first marketed for intravenous (IV) administration, but recently a formulation for subcutaneous (SC) use has been made available.^2^ SC formulation of daratumumab proved to be non‐inferior in comparison with IV administration route in terms of efficacy, pharmacokinetics, and safety in the Phase 3 COLUMBA trial.^3^, ^4^

Intravenous administration route implies relevant consumption of non‐pharmacological health‐care resources, like nursing time, infusion chairs, and disposables.

Several studies in oncology found that administering the same drug subcutaneously instead of intravenously results in a plethora of advantages. In particular, rituximab and trastuzumab, for which both formulations are available, were studied. With respect to IV infusions, SC administrations may result in economic savings,^5^, ^6^, ^7^, ^8^ reduction in time spent by health care professionals^5^, ^7^, ^8^, ^9^, ^10^ and patients,^6^, ^7^, ^8^, ^9^ reduction in mean patient chair time,^5^, ^9^, ^10^ improvement in the health‐related quality of life (HRQoL),^5^ and decrease in wastage.^7^ In addition, patients' generally prefer SC over IV formulations.^5^, ^6^ It should also be considered that the time saved by patients and their caregivers is responsible for the increase in their productivity.^6^ Healthcare professional (HCP) time saved may be invested in other activities, thereby increasing their productivity.^9^ The chair time saved allows to arrange a greater number of appointments, reduce waiting lists, and increase the Oncology Unit efficiency.^9^

In the literature search conducted by the group of Anderson about the previous 5 years, 8 articles investigated the differences between the administration routes by using the time‐and‐motion methodology, that allow HCPs to identify all the relevant tasks undertaken during the processes and to collect time actively spent by HCPs and patients in each of them, also quantifying the chair time.

This study aimed at assessing the economic impact associated with the use of SC versus IV daratumumab in patients with RRMM from the perspective of the hematology center.

## 2 METHODS

### 2.1 Main characteristics of the study

This multicenter cross‐sectional micro‐costing study was carried out from June 27, 2022, to November 30, 2022 in three Italian hematology centers.

The inclusion criteria were: age ≥18 years old; RRMM diagnosis; ongoing treatment with IV or SC daratumumab; access to the clinical center due to a previously scheduled daratumumab administration; willingness to participate in the study by signing the informed consent form.

The primary endpoint was the comparison of the mean cost of IV and SC administrations of daratumumab in patients with RRMM from the hematology center perspective. The following costs were taken into account: healthcare resources consumed (drugs and consumables), active working time spent by the HCPs, and use of durable equipment.

The secondary endpoints were: (1) HCP time, that is, the evaluation of the HCP active working time, including total time, time per every HCP role and per each task; (2) patient total time, that is, the evaluation of patient's length of stay in the hematology center for the administration; it was calculated as the total time between the material preparation and the infusion field cleaning; and (3) chair total time, that is, the evaluation of time spent for durable equipment use (infusion chair or bed).

### 2.2 Study entry and measurements

While accessing the hematology center for a scheduled daratumumab administration, patients were made aware of the possibility to enter this study. After the explanation of the design and purpose of the study by clinicians or nurses, patients were given written information and, if willing to participate, they signed the informed consent that authorized to process their personal data.

Measurements were taken on site directly by HCPs involved in the preparation, administration, and monitoring of the study drug thanks to the EASIER App. It was installed on the mobile phone of each HCP involved in the study. The App measured the selected procedure or task by means of start/stop buttons. Resources used for the procedure were associated directly. HCPs were allowed to use the App concurrently. Due to the cross‐sectional nature of the study, data were collected just once and there was no follow‐up.

The procedures we followed were in accordance with the ethical standards of the responsible committee on human experimentation and with the Helsinki Declaration of 1975, as revised in 2013. The study was approved by the ethics committees of each center involved.

The sample size was judged sufficient to carry out a health economics analysis.

Table S1 shows the macro‐tasks that were considered when measuring the steps of the procedure by means of the EASIER App and how they were aggregate to perform the analysis.

### 2.3 Statistical analyses

When data were lacking, the mean value of the lacking task estimated in the same center was input.

The categorical variables were represented in terms of absolute and relative frequencies (percentages), while the continuous variables were synthesized by showing mean, standard deviation and, if deemed informative, total range (min–max).

To account for the possible differences about the organization and the resource value among the participating centers, total costs were stratified by center and the total impact was estimated by weighting the cost estimated in each center by the proportion of patients enrolled.

### 2.4 Health economics analyses

A pertinent unit cost, gathered directly from the accounting departments of participating centers, was assigned to every resource consumed.

The cost of the working time of HCPs was valued according to the opportunity‐cost principle and valorized according to the center‐specific cost per hour.

The depreciation cost that was assigned to the use of durable equipment came from a single center: this value was applied also for the others, as specific inputs were lacking.

The consumption of the materials that were necessary for the drug administration was valued by using the unit costs collected during the study.

In accordance with the good activity‐based accounting practice,^6^ hospital general costs, including cost items nonspecific of the department where the drug was administered, have to be considered in the total cost. Hospital general costs were reversed *pro quota* on the center according to the activity provided. Intermediate healthcare services, common costs, general and administrative costs, depreciation, purchases of non‐healthcare goods, and services were included. For this purpose, 25% was added to the intermediate calculation that was obtained, based on the general estimate that hospital general costs represent 20% of the total costs of a healthcare service.^6^

## 3 RESULTS

Ninety‐five daratumumab administration procedures were measured—19 (20%) IV and 76 (80%) SC. IV and SC groups were unbalanced as, when the measurements were performed, the switch from IV to SC formulation had already been going on for some weeks and thus had already involved most of the patients treated with daratumumab. The number of procedures measured per center ranged from 27 to 40.

### 3.1 Patient time

Table 1 shows that patients spent on average almost 5 h in the hematology center to receive the IV administration of daratumumab and a just over 1 h to receive the SC administration.

**TABLE 1:** Patient time measured in the study population.

Patient time (h)	IV daratumumab	SC daratumumab	Δ IV vs. SC daratumumab (95% CI)^a^
No. of procedures	16	66	
Mean ± SD	4.85 ± 0.91	1.08 ± 0.56	3.77 (3.4–4.1)
Median (IQR)	4.4 (4.2–5.5)	1.1 (0.6–1.4)	
Range	3.8; 6.5	0.2; 2.9	

Abbreviations: CI, confidence interval; IQR, interquartile range; IV, intravenous; SC, subcutaneous; SD, standard deviation.

^a^ Estimated with linear regression.

The relevant box and whiskers plot is shown in Figure S1.

On average, each SC procedure reduced the time spent by patients in the hematology center by 3.8 h (95% CI 3.4–4.1). Therefore, SC route saved on average 78% of patient time, if compared to IV administration.

### 3.2 HCP active working time

HCPs, that is, nurses, pharmacists, and physicians, devoted more active working time for IV than for SC procedures, before, during, and after the actual administration (Table 2).

**TABLE 2:** HCP active working time measured in the study population.

HCP active working time (min)	IV daratumumab	SC daratumumab	Δ IV vs. SC daratumumab (95% CI)^a^
Pre‐infusion			
*N*	19	76	
Mean ± SD	27.57 ± 9.33	10.01 ± 6.74	17.6 (13.8–21.3)
Median (IQR)	29 (18.9–33.6)	9.2 (4.2–14)	
Range	11.1; 44.6	1.8; 36	
Infusion			
*N*	17	69	
Mean ± SD	15.22 ± 19.7	5.92 ± 1.93	9.3 (4.6–14)
Median (IQR)	4.7 (2.2–20)	5.9 (4.6–6.9)	
Range	0; 62.8	1.1; 12.9	
Post‐infusion			
*N*	18	66	
Mean ± SD	9.03 ± 7.61	4.41 ± 6.02	4.6 (1.2–8)
Median (IQR)	6.4 (2.9–14.8)	1.5 (0.8–6.5)	
Range	1.5; 27	0.4; 33.5	
Total			
*N*	16	66	
Mean ± SD	49.38 ± 16.13	20.37 ± 7.88	29.0 (23.5–34.5)
Median (IQR)	46.2 (37.1–62.2)	19.5 (15.1–24.8)	
Range	21.4; 78.1	5.7; 42.3	

Abbreviations: CI, confidence interval; HCP, healthcare professional; IQR, interquartile range; IV, intravenous; N, number of patients with data; SC, subcutaneous; SD, standard deviation.

^a^ Estimated with linear regression.

Globally, mean HCP active working time per procedure was 49 and 20 min for IV and SC administrations, respectively. Time ratios between the administration routes are constant for each of the three phases of administration, as the active working time of IV administration is two‐ to threefold than the active time of SC administration in each of the 3 phases: 28 versus 10 min (−64%) in the pre‐infusion phase, 15 versus 6 min (−60%) in the infusion phase, and 9 versus 4 min (−55%) in the post‐infusion phase.

Therefore, the additional HCP active working time for each IV versus SC administration was about half an hour (29 min, 95% CI 23.5 to 34.5), of which 55.8% was avoided in pre‐infusion phase (17.6 min; 95% CI 13.8 to 21.3), 29.5% during infusion (9.3 min; 95% CI 4.6 to 14), and 14.7% in post‐infusion phase (4.6 min; 95% CI 1.2 to 8). The relevant box and whiskers plots according to daratumumab administration phases are shown in Figure S2.

It should be noticed that SC administration allowed HCPs to save roughly 59% of their time required for IV infusion. Among HCPs, the prominent role was played by the nurse, whose time accounted for 79% of the total working time, while the pharmacist and the physician devoted 19% and 2% of the total working time, respectively.

### 3.3 Chair time

Healthcare resources in terms of chair time for the administration of daratumumab were also measured, as reported in Table 3.

**TABLE 3:** Chair time measured in the study population.

Chair time (h)	IV daratumumab	SC daratumumab	Δ IV vs. SC daratumumab (95% CI)^a^
No. of procedures	16	66	
Mean ± SD	4.85 ± 0.91	0.99 ± 0.55	3.86 (3.5–4.2)
Median (IQR)	4.4 (4.2–5.5)	0.9 (0.6–1.4)	
Range	3.8; 6.5	0.2; 2.9	

Abbreviations: CI, confidence interval; IQR, interquartile range; IV, intravenous; SC, subcutaneous; SD, standard deviation.

^a^ Esti mated with linear regression.

The infusion chair/bed was occupied on average for 4.85 h during IV infusion and 0.99 h for SC administration, thereby delineating a considerable reduction in chair time with SC administration. In fact, occupancy time for SC administration was on average 3.86 h shorter (95% CI 3.5 to 4.2) than IV procedure, resulting in 80%‐time decrease (Table 2). The relevant box and whiskers plot is shown in Figure S3.

### 3.4 Costs

We considered the lists of materials used for IV and SC administrations in every participating hematology center and the relevant costs (according to each center), together with the costs due to the time spent by HCPs to administer the drug (including pre‐infusion, infusion and post‐infusion time) and the chair occupancy. Therefore, we calculated the direct healthcare costs borne by each hematology center for each hospital administration procedure of daratumumab, according to the administration route and estimated absolute difference (Table 4).

**TABLE 4:** Direct healthcare costs borne by each hematology center for each hospital administration procedure of daratumumab, according to the administration route. HCP time includes pre‐infusion, infusion, and post‐infusion time.

Costs (€)	IV daratumumab (mean ± SD)	SC daratumumab (mean ± SD)	Δ IV vs. SC daratumumab (95% CI)^b^	% Costs saved with SC daratumumab^b^
Total cost^a^ (Center 1)	103.74 ± 32.99	48.31 ± 21.42	55.43 (27.15–83.72)	−53%
HCP time	23.27 ± 3.07	6.70 ± 2.79	16.57 (13.03–20.10)	−71%
Chair time	0.49 ± 0.04	0.10 ± 0.02	0.39 (0.36–0.42)	−80%
Materials	59.24 ± 27.03	31.85 ± 15.88	27.39 (6.03–48.75)	−46%
Total cost^a^ (Center 2)	45.60 ± 8.34	20.92 ± 6.51	24.68 (19.28–30.07)	−54%
HCP time	22.31 ± 6.38	10.59 ± 5.21	11.72 (7.67–15.78)	−53%
Chair time	0.36 ± 0.06	0.07 ± 0.06	0.29 (0.25–0.34)	−81%
Materials	14.16 ± 4.25	6.08 ± 1.83	8.08 (4.12–12.04)	−57%
Total cost^a^ (Center 3)	106.71 ± NA	42.07 ± 15.39	64.64 (31.63–97.64)	−61%
HCP time	35.44 ± NA	13.93 ± 6.87	21.42 (6.75–36.08)	−60%
Chair time	0.44 ± NA	0.07 ± 0.04	0.37 (0.29–0.44)	−84%
Materials	49.59 ± NA	19.36 ± 7.83	30.23 (13.75–46.71)	−61%

Abbreviation: NA, not available as only one patient performed IV administration.

^a^ Includes general hospital costs.

^b^ Estimated with linear regression.

**FIGURE 1:**
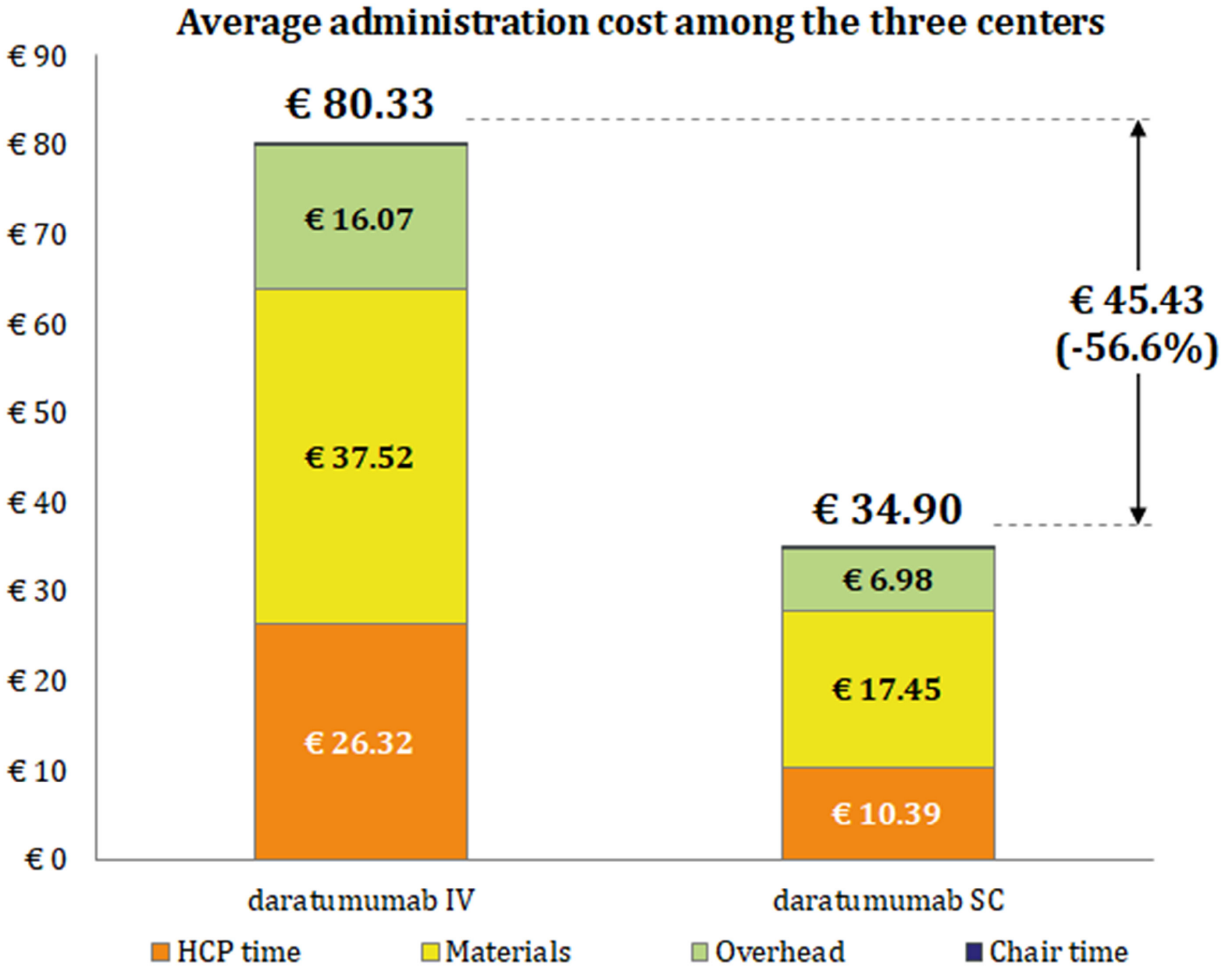
Mean cost per daratumumab administration.

Including overhead costs, among the three centers, each SC daratumumab administration was associated with savings of about €45 (−57%) when compared to its corresponding IV infusion (Figure 1).

Most of this reduction was due to the decrease in the active working time spent by HCPs to administer the drug (−60.4%). The saving was due also to the decrease in the consumption of healthcare materials and the use of equipment, as well as the shorter stay at the center (−53.5%). The cost due to chair time, on the other hand, had very low impact (less than one euro per administration). The reduction due to SC administration was overall higher than 80%.

## 4 DISCUSSION

Daratumumab proved its efficacy and safety in patients affected by multiple myeloma.^11^, ^12^, ^13^, ^14^, ^15^, ^16^, ^17^, ^18^ Treatment regimens including daratumumab are currently the standard of care for newly diagnosed patients, whether eligible and ineligible to transplant.^19^ In RRMM setting, daratumumab is approved both as monotherapy and in combination with proteasome inhibitors and immunomodulators.^1^

However, long infusion times and infusion‐related adverse events may affect patients' quality of life and burden of the healthcare service.^2^, ^4^, ^20^ In order to try and overcome these issues, a SC formulation was developed, in which 1800 mg daratumumab were co‐formulated with 2000 IU/mL recombinant human hyaluronidase PH20.^4^ While in IV formulation dosing has to be adjusted according to patients' weight, SC daratumumab is administered as a flat dose, thus simplifying also drug preparation.^21^ COLUMBA trial was a multicenter, open‐label, randomized, Phase 3 study that proved the non‐inferiority of SC daratumumab versus IV daratumumab in terms of efficacy, pharmacokinetics (maximum *C*_trough_, that is strongly correlated with efficacy,^3^) and safety both in the first analysis, at 7.5 months of follow‐up,^4^ and in the final analysis at 29.3 months of follow‐up.^3^

The present study found that, by administering daratumumab subcutaneously instead of intravenously, it was possible to spare 78%, 59%, 80% of patient time, HCP active working time, and infusion chair time, respectively. From an economic point of view, on average 56.6% of total expenses, corresponding to €45.43, would be spared per every administration, as a result of the savings coming from HCP and chair time spared, overhead costs, and the costs for materials used during infusion.

Far from being an advantage only from an economical point of view, the use of SC instead of IV daratumumab may result in great organizational advantages for the whole hematology wards, that reverberates also on patients treated with regimens other than daratumumab. In fact, the decreased occupancy time of infusion chairs allows other patients to use them, thus potentially resulting in waiting lists shortening, in accordance with observations by other authors comparing SC versus IV administration.^9^, ^22^ It may be beneficial also from environmental impact point of view, due to the lower quantity of waste produced by the SC administration vs the IV one.^7^

The time spent by HCPs deserves some considerations. Their active working time is not directly proportional to the infusion length, as their presence is surely required at the beginning and at the end of the administration, while during it they come by every now and then to check. There are some pre‐set timepoints to change the infusion rate after checking the patient’ vital signs. A close monitoring is required in case of adverse reactions or reported symptoms. In this study, time ratios between the administration routes are constant: the active working time of IV administration is two‐ to threefold than the active time of SC administration in each of the three phases (28, 15, and 9 min IV compared with 10, 6, and 4 min SC for pre‐infusion, infusion, post‐infusion phases, respectively). The greatest impact is in the pre‐infusion phase, as the absolute time spared with SC administration during the pre‐infusion phase is greater than the time spared during the infusion phase. This is due to the greatest effort required from HCPs in the pre‐infusion procedures, due to the time needed to dilute daratumumab to be administered intravenously (conversely, SC formulation is ready to use).

The time spared by HCPs may be reinvested in other activities and enhance the ward organization^9^: this possibility is particularly welcomed due the increasingly worrying shortage of HCPs in the Italian HealthCare Service.^23^

It should also be considered that the less the time spent in hospital, the less the likelihood of acquiring a Health‐Care Associated Infection: this is particularly important, due to the immunological fragility of onco‐hematological patients.^24^ In these years of COVID‐19 pandemic, the reduction of length of stays in hospital has been particularly important in order to reduce and prevent the risk of infections.

Data coming from COLUMBA study^25^ suggest that patients' satisfaction is greater when receiving daratumumab subcutaneously than intravenously. Together with clinical efficacy, that was shown to be non‐inferior than IV formulation, patients satisfaction with SC daratumumab may enhance patients' adherence to therapy,^26^, ^27^, ^28^, ^29^, ^30^ that is particularly important in the oncology setting, where long‐term treatments are required to gain control on the disease. Caregiver time, also, would be likely reduced, as reported by Slavcev and colleagues.^24^

Among the strengths of this research, there is the real‐world collection of data. In particular, the time‐and‐motion design of the study allows a precise calculation of the time spent in each task. This type of study was born at the beginning of the last century as a merge of time study and motion study, where the first was meant to measure, by means of stopwatch observations, the time spent by people performing a range of occupations, while the second aimed at minimizing the number of moves required to finish a task, thus improving efficiency.^31^ Initially applied to industrial engineering, it has subsequently been largely used by biological and clinical researchers due to its cost‐saving potential.^31^ Basically, a difficult task is divided in smaller steps, which are measured in terms of time spent and the results are then analyzed to identify and eliminate unnecessary motions.^31^

Other time‐and‐motion studies have been conducted in oncology setting regarding the switch from IV to SC formulations of rituximab and trastuzumab, as reported by Anderson and colleagues^5^: all the 8 studies identified by this study group concluded that economic savings were associated with the switch from IV to SC. They found a reduction in HCP active working time ranging from 31% and 79% (13–49 min and 27–223 min for SC and IV formulations, respectively) and in chair time ranging from 74% and 85% (11–70 min and 75–264 min for SC and IV formulations, respectively). Our findings (59% reduction in HCP working time and 80% reduction in infusion chair time) fall in the middle of these ranges. To the best of our knowledge, this is the first time‐and‐motion study comparing SC and IV daratumumab.

However, other studies have gathered data about this switch. Slavcev and colleagues^24^ carried out a web‐based time‐and‐motion survey that was completed by 26 HCPs at sites involved in the enrollments in the COLUMBA trial. Qualitative and quantitative opinion‐based estimates about HCP active working time were collected. Not considering the first administration, for which more time is dedicated for monitoring purposes, the median total HCP active working time was reduced by 49.5% (179.2 min vs. 90.4 min in IV and SC daratumumab administration, respectively). The estimated chair time decreased by 97% (238.0 min vs. 8.1 min in IV and SC administration routes, respectively). Administration duration was the key driver in total reduction of patient chair occupation. It should be pointed out that as reported in Table S1, in our study, chair time, that lasts definitely almost 1 h, includes also the cleaning, waste disposal, relocation of unused material, and sometimes monitoring (if performed on the infusion chair) and, in brief, any activity that prevents others from using the infusion chair, even though the patients is not actively occupying it.

In the study conducted by Soefje et al.,^32^ time‐based measurements were extracted from electronic records using a scheduling/pharmacy software program. They found that, when using SC daratumumab, patient time was reduced by 2.7–3.0 h (it was 4.8 h for IV infusion) and median total chair time was 2.7–2.8 h shorter (it was 4.0 h for IV infusion) with respect to IV daratumumab. Due to the design of the study, the estimations were performed differently to our time‐and‐motion study: patient time was calculated as the difference between the time at patient check‐in and the time at patient check‐out and the chair time was estimated as the difference between the time from infusion room entry and the time of infusion room exit.

An interesting study carried out by Federici and colleagues^22^ conducted semi‐structured interviews to six onco‐hematologists coming from Italian Cancer Centers about organization and time spent to administer IV and SC daratumumab to populate a Discrete‐Event Simulation (DES) model. It aimed at analyzing the impact of the variations of incident patients requiring IV or SC daratumumab in terms of ward organization. They demonstrated that, differently from SC daratumumab, if IV daratumumab was administered, the ward would not be able to absorb the impact of more‐than‐expected cancer patients (for 20 instead of 3 additional patients per week, 17 additional infusion chairs would be required). The estimates gathered by experts differ in some assumptions by our study. While pre‐infusion HCP active working time in IV route was similar (29 vs. 28 min in our study), pre‐infusion SC time was different (0 vs. 10 min), as Federici and colleagues considered that prefilled syringe did not require preparation, while we included in that time also the necessary measurement of patients' parameters such as temperature, blood pressure, heart rate, etc. Again, HCP active time during infusion was similar for SC administration (5 vs. 6 min), but different for IV infusion (48 vs. 15 min), post‐infusion times were different (23 vs. 4 min for SC and 23 vs. 9 min for IV), as also chair occupancy time (5 vs. 59 min for SC infusion vs. 3.15 vs. 4.8 h for IV infusions). The latter may depend on our method to calculate chair time, that was already described above.

This work has also some limitations. The sample was unbalanced, as in two centers the number of IV administrations was very low. This was due to bureaucratic delays, that resulted in the beginning of the measurements when the SC administration was already reimbursed. Therefore, almost all patients were already being administered subcutaneously. In fact, as suggested by a group of UK experts in MM (UK was an early adopter of SC daratumumab), hematologists believe that most patients are willing to use SC instead of IV daratumumab, with some possible exceptions, like patients who have achieved disease control with IV daratumumab after several previous attempts with other drugs, needle‐phobic patients, and patients affected by specific skin conditions or significant edema.^21^

In addition, we analyzed three centers in three different regions in the Northern Italy (Piemonte, Lombardia, and Veneto). As every region has its HealthCare Service, another limitation comes from the differences found among centers in terms of absolute values of costs, even though the saving proportion are similar both considering total costs and costs coming from each cost item. In fact, Center 1, 2 and 3 spare €55.43, €24.68, and €64.64, respectively. We can conclude that presumably in all the Italian regions the total expense for every administration would more than halve, but uncertainty exists about the absolute savings in euros.

We underline also that daratumumab is frequently administered in association with other SC or IV drugs, such as bortezomib and dexamethasone, that may have an effect on the overall chair time and patient time.

Any possible difference in terms of frequency or type of adverse events (AE) between IV and SC formulations would affect time and costs calculations, but we detected no AE, maybe due to the low sample size. We chose not to include the first administrations because, as reported in the relevant Summary of Product Characteristics, the IV formulation is burdened by longer time of administration and higher frequency of adverse events than the subsequent administrations. As a consequence, the difference between IV and SC administrations would have been further amplified. Therefore, we adopted a conservative approach. In general, it is worth considering that the reduced incidence and entity of infusion reactions with SC formulation may have an impact on the way the premedication is performed, thus potentially affecting not only pre‐infusion, but also post‐infusion time, should a post‐medication be administered in at‐risk individuals.

In conclusion, this time‐and‐motion study performed real‐world measurements that showed that SC administration of daratumumab is associated with consistent savings in terms of time and budget as compared with IV administration. In particular, 78%, 59%, 80% of patient time, HCP active working time, and infusion chair time, respectively, and 56.6% of total expenses are spared. We expect that this switch results in further advantages, such as better hematology ward organization, enhanced patient satisfaction, compliance to treatment and well‐being, reduced waste, and decreased waiting lists also for other treatments requiring infusion chair.

## AUTHOR CONTRIBUTIONS

**Lorenzo Pradelli:** Conceptualization (lead); formal analysis (lead); methodology (lead); supervision (lead); writing – original draft (lead). **Massimo Massaia:** Investigation (equal); validation (equal); writing – review and editing (equal). **Elisabetta Todisco:** Investigation (equal); validation (equal); writing – review and editing (equal). **Filippo Gherlinzoni:** Investigation (equal); validation (equal); writing – review and editing (equal). **Anna Furlan:** Investigation (equal); validation (equal); writing – review and editing (equal). **Maria La Targia:** Investigation (equal); validation (equal); writing – review and editing (equal). **Elisabetta Grande:** Investigation (equal); validation (equal); writing – review and editing (equal). **Ignazio Ezio Tripoli:** Investigation (equal); validation (equal); writing – review and editing (equal). **Francesca Occhipinti:** Investigation (equal); validation (equal); writing – review and editing (equal). **Francesco Comello:** Investigation (equal); validation (equal); writing – review and editing (equal). **Fabrizio Iannello:** Investigation (equal); validation (equal); writing – review and editing (equal). **Stefania Bellucci:** Investigation (equal); validation (equal); writing – review and editing (equal).

## CONFLICT OF INTEREST STATEMENT

LP is the co‐owner and an employee of AdRes, which has received project funding from Janssen‐Cilag SpA. MM reports advisory boards for AbbVie, Janssen‐Cilag, Sanofi, and research funding from Sanofi. FO received speaker fees from Bio‐Rad Laboratories. ET, FG, AF, MLT, EG, IET, and FC have no other competing interests outside the abovementioned funding of this study. FI and SB are employees at Janssen‐Cilag.

## ETHICS STATEMENT

This study was approved by the following ethics committees: Comitato Etico dell'Insubria, Comitato Etico per le Sperimentazioni Cliniche (CESC) delle Province di Treviso e Belluno, and Comitato Etico Interaziendale dell'A.O. S. Croce e Carle di Cuneo – ASL CN1 – ASL CN2 – ASL AT.

## PATIENT CONSENT STATEMENT

Patients were given written information and, if willing to participate, they signed the informed consent that authorized to process their personal data.

## ETHICS AND INTEGRITY POLICY

The procedures we followed were in accordance with the ethical standards of the responsible committee on human experimentation and with the Helsinki Declaration of 1975, as revised in 2013.

## Supporting information


Table S1.
Figure S1.
Figure S2.

## Data Availability

The data that support the findings of this study are available from the corresponding author upon reasonable request.

## References

[1] AIOM . Linee guida Mieloma. AIOM. 2017. Accessed March 14, 2023. https://www.aiom.it/mieloma‐2017/

[2] European Medicines Agency . Darzalex. Summary of product characteristics. 2022. Accessed March 14, 2023 https://www.ema.europa.eu/en/documents/product‐information/darzalex‐epar‐product‐information_en.pdf

[3] Usmani SZ , Nahi H , Legiec W , et al. Final analysis of the phase III non‐inferiority COLUMBA study of subcutaneous versus intravenous daratumumab in patients with relapsed or refractory multiple myeloma. Haematologica. 2022;107(10):2408‐2417. doi:10.3324/haematol.2021.279459 35354247 PMC9521240

[4] Mateos MV , Nahi H , Legiec W , et al. Subcutaneous versus intravenous daratumumab in patients with relapsed or refractory multiple myeloma (COLUMBA): a multicentre, open‐label, non‐inferiority, randomised, phase 3 trial. Lancet Haematol. 2020;7(5):e370‐e380. doi:10.1016/S2352-3026(20)30070-3 32213342

[5] Anderson KC , Landgren O , Arend RC , Chou J , Jacobs IA . Humanistic and economic impact of subcutaneous versus intravenous administration of oncology biologics. Future Oncol. 2019;15(28):3267‐3281. doi:10.2217/fon-2019-0368 31394933

[6] Cicchetti A , Coretti S , Mascia D , et al. Assessing social and economic impact of subcutaneous mAbs in oncology. Glob Re Health Technol Assess. 2018;5(1):1‐9. doi:10.33393/grhta.2018.443

[7] Ponzetti C , Canciani M , Farina M , Era S , Walzer S . Potential resource and cost saving analysis of subcutaneous versus intravenous administration for rituximab in non‐Hodgkin's lymphoma and for trastuzumab in breast cancer in 17 Italian hospitals based on a systematic survey. Clinicoecon Outcomes Res. 2016;8:227‐233. doi:10.2147/CEOR.S97319 27284260 PMC4883807

[8] Rule S , Collins GP , Samanta K . Subcutaneous vs intravenous rituximab in patients with non‐Hodgkin lymphoma: a time and motion study in the United Kingdom. J Med Econ. 2014;17(7):459‐468. doi:10.3111/13696998.2014.914033 24720836

[9] De Cock E , Kritikou P , Sandoval M , et al. Time savings with rituximab subcutaneous injection versus rituximab intravenous infusion: a time and motion study in eight countries. PloS One. 2016;11(6):e0157957. doi:10.1371/journal.pone.0157957 27362533 PMC4928781

[10] De Cock E , Pivot X , Hauser N , et al. A time and motion study of subcutaneous versus intravenous trastuzumab in patients with HER2‐positive early breast cancer. Cancer Med. 2016;5(3):389‐397. doi:10.1002/cam4.573 26806010 PMC4799946

[11] Lokhorst HM , Plesner T , Laubach JP , et al. Targeting CD38 with daratumumab monotherapy in multiple myeloma. N Engl J Med. 2015;373(13):1207‐1219. doi:10.1056/NEJMoa1506348 26308596

[12] Moreau P , Hulin C , Perrot A , et al. Maintenance with daratumumab or observation following treatment with bortezomib, thalidomide, and dexamethasone with or without daratumumab and autologous stem‐cell transplant in patients with newly diagnosed multiple myeloma (CASSIOPEIA): an open‐label, randomised, phase 3 trial. Lancet Oncol. 2021;22(10):1378‐1390. doi:10.1016/S1470-2045(21)00428-9 34529931

[13] Facon T , Kumar S , Plesner T , et al. Daratumumab plus lenalidomide and dexamethasone for untreated myeloma. N Engl J Med. 2019;380(22):2104‐2115. doi:10.1056/NEJMoa1817249 31141632 PMC10045721

[14] Mateos MV , Dimopoulos MA , Cavo M , et al. Daratumumab plus bortezomib, melphalan, and prednisone for untreated myeloma. N Engl J Med. 2018;378(6):518‐528. doi:10.1056/NEJMoa1714678 29231133

[15] Palumbo A , Chanan‐Khan A , Weisel K , et al. Daratumumab, bortezomib, and dexamethasone for multiple myeloma. N Engl J Med. 2016;375(8):754‐766. doi:10.1056/NEJMoa1606038 27557302

[16] Dimopoulos MA , Oriol A , Nahi H , et al. Overall survival with daratumumab, lenalidomide, and dexamethasone in previously treated multiple myeloma (POLLUX): a randomized, open‐label, phase III trial. J Clin Oncol. 2023;41(8):1590‐1599. doi:10.1200/JCO.22.00940 36599114 PMC10022849

[17] Lonial S , Weiss BM , Usmani SZ , et al. Daratumumab monotherapy in patients with treatment‐refractory multiple myeloma (SIRIUS): an open‐label, randomised, phase 2 trial. Lancet. 2016;387(10027):1551‐1560. doi:10.1016/S0140-6736(15)01120-4 26778538

[18] Usmani SZ , Weiss BM , Plesner T , et al. Clinical efficacy of daratumumab monotherapy in patients with heavily pretreated relapsed or refractory multiple myeloma. Blood. 2016;128(1):37‐44. doi:10.1182/blood-2016-03-705210 27216216 PMC4937359

[19] Offidani M , Corvatta L , Morè S , et al. Daratumumab for the Management of Newly Diagnosed and Relapsed/refractory multiple myeloma: current and emerging treatments. Frontiers in Oncology. 2021;10:1‐14. doi:10.3389/fonc.2020.624661 PMC7928404 33680948

[20] Bittner B , Richter W , Schmidt J . Subcutaneous Administration of Biotherapeutics: an overview of current challenges and opportunities. BioDrugs. 2018;32(5):425‐440. doi:10.1007/s40259-018-0295-0 30043229 PMC6182494

[21] Cook G , Ashcroft J , Fernandez M , et al. Benefits of switching from intravenous to subcutaneous daratumumab: Perspectives from UK healthcare providers. Front Oncol. 2023;13:1063144. doi:10.3389/fonc.2023.1063144 36910662 PMC9996301

[22] Federici C , Rognoni C , Costa F , Armeni P , Crovato E , Bellucci S . Use of resource modeling to quantify the organizational impact of subcutaneous formulations for the treatment of oncologic patients: the case of daratumumab in multiple myeloma. Clin Ther. 2022;44(11):1480‐1493. doi:10.1016/j.clinthera.2022.09.006 36195503

[23] Il Sole 24 ore . Cittadinanzattiva: la carenza di personale attraversa tutta l'Italia, si rischia il deserto sanitario. *Sanità24* . 2023. Accessed July 26, 2023 http://s24ore.it/loD3SU

[24] Slavcev M , Spinelli A , Absalon E , et al. Results of a time and motion survey regarding subcutaneous versus intravenous administration of daratumumab in patients with relapsed or refractory multiple myeloma. Clinicoecon Outcomes Res. 2021;13:465‐473. doi:10.2147/CEOR.S302682 34135605 PMC8197571

[25] Usmani SZ , Mateos MV , Hungria V , et al. Greater treatment satisfaction in patients receiving daratumumab subcutaneous vs. intravenous for relapsed or refractory multiple myeloma: COLUMBA clinical trial results. J Cancer Res Clin Oncol. 2021;147(2):619‐631. doi:10.1007/s00432-020-03365-w 32852632 PMC11801943

[26] Revicki DA . Patient assessment of treatment satisfaction: methods and practical issues. Gut. 2004;53 Suppl 4(Suppl 4):iv40‐iv44. doi:10.1136/gut.2003.034322 15082613 PMC1867784

[27] Sweileh WM , Ihbesheh MS , Jarar IS , et al. Self‐reported medication adherence and treatment satisfaction in patients with epilepsy. Epilepsy Behav. 2011;21(3):301‐305. doi:10.1016/j.yebeh.2011.04.011 21576040

[28] Price D , Harrow B , Small M , Pike J , Higgins V . Establishing the relationship of inhaler satisfaction, treatment adherence, and patient outcomes: a prospective, real‐world, cross‐sectional survey of US adult asthma patients and physicians. World Allergy Organ J. 2015;8(1):26. doi:10.1186/s40413-015-0075-y 26417397 PMC4564954

[29] Small M , Anderson P , Vickers A , Kay S , Fermer S . Importance of inhaler‐device satisfaction in asthma treatment: real‐world observations of physician‐observed compliance and clinical/patient‐reported outcomes. Adv Ther. 2011;28(3):202‐212. doi:10.1007/s12325-010-0108-4 21331556

[30] Barbosa CD , Balp MM , Kulich K , Germain N , Rofail D . A literature review to explore the link between treatment satisfaction and adherence, compliance, and persistence. Patient Prefer Adherence. 2012;6:39‐48. doi:10.2147/PPA.S24752 22272068 PMC3262489

[31] Kalne PS , Mehendale AM . The purpose of time‐motion studies (TMSs) in healthcare: a literature review. Cureus. 2022;14(10):e29869. doi:10.7759/cureus.29869 36348835 PMC9629289

[32] Soefje SA , Carpenter C , Carlson K , et al. Clinical administration characteristics of subcutaneous and intravenous Administration of Daratumumab in patients with multiple myeloma at Mayo Clinic infusion centers. JCO Oncol Pract. 2023; 19(4):e542‐e549. doi:10.1200/OP.22.00421 PMC10101255 36758192

