# Supplementary material for: Improved efficiency of daratumumab treatment of multiple myeloma adopting the subcutaneous route: A micro‐costing analysis in three Italian hematology centers
Source: Cancer Med. 2023 Nov 9;12(23):21480–9. doi: 10.1002/cam4.6699 (PMC10726826; doi:10.1002/cam4.6699)
Supplement: Supplementary file 1 — Table S1. Figure S1. Figure S2. [file CAM4-12-21480-s001.docx]

| **Time** | **Tasks** | **Description** |
| --- | --- | --- |
| Pre-infusion time | 1. Material preparation | Material pick-up, placement next to the infusion chair. It doesn’t include the drug pick-up |
|  | 1. Drug pick-up at the hospital pharmacy | Round trip to the hospital pharmacy, drug pick-up, placement in the ward refrigerator |
|  | 1. Patient preparation | The patient undresses/gets dressed, measurement of parameters such as temperature, blood pressure, heart rate, etc.; possible visit, seating on chair/bed, possible pre-dressing, disinfection, and venous access (in case of IV administration) |
|  | 1. Drug preparation | Pick-up, dilution (in case of IV administration), visual inspection |
| Infusion time | 1. Drug administration—active time | **IV administration**: infusion set attachment, outflow connection to the catheter needle, additional active time dedicated to the patient during the infusion (routine control or upon request)  **SC administration**: ready-to-use disposable, preparation of the syringe under controlled and validated aseptic conditions, connection of the hypodermic needle for injections or the SC infusion set to the syringe immediately before injection |
| Post-infusion time | 1. Post-infusion tasks | Intravenous flushing, set removal (in case of IV infusion), dressing, clearing of infusion chair/bed (if monitoring elsewhere) |
|  | 1. Monitoring—Active Time | Accommodating the patient in the monitoring site (if different from the administration site), additional active time dedicated to the patient during monitoring for routine check-ups or upon request, patient discharge |
|  | 1. Infusion field cleaning and waste disposal | Cleaning of the infusion chair/bed, waste disposal, relocation of unused material. Please note: write down the armchair/bed among the materials used if they cannot be used by other patients during cleaning |

Supporting Table S1. Macro-tasks for each procedure.

H = hospital; HCP = healthcare professional; IV = intravenous; SC = subcutaneous


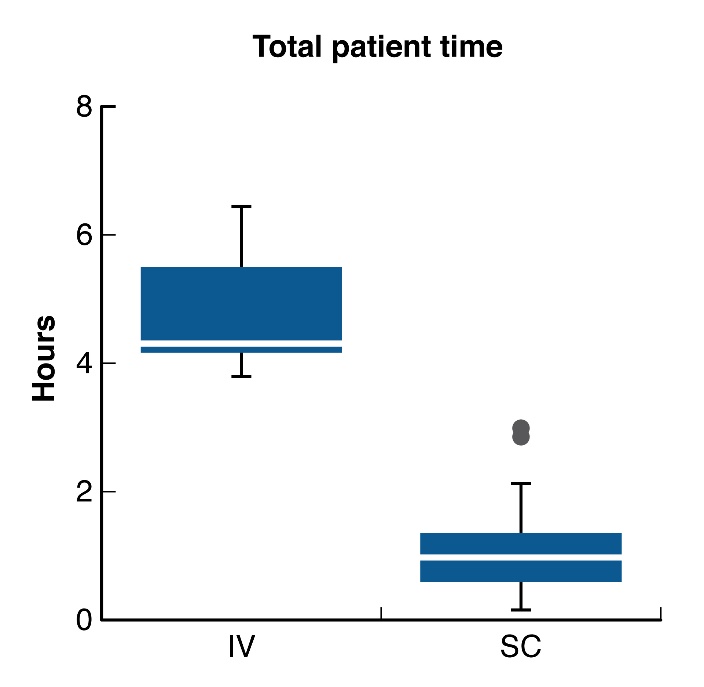


Supporting Figure S1. Box and whiskers plot relative to patient times.

IV = intravenous; SC = subcutaneous


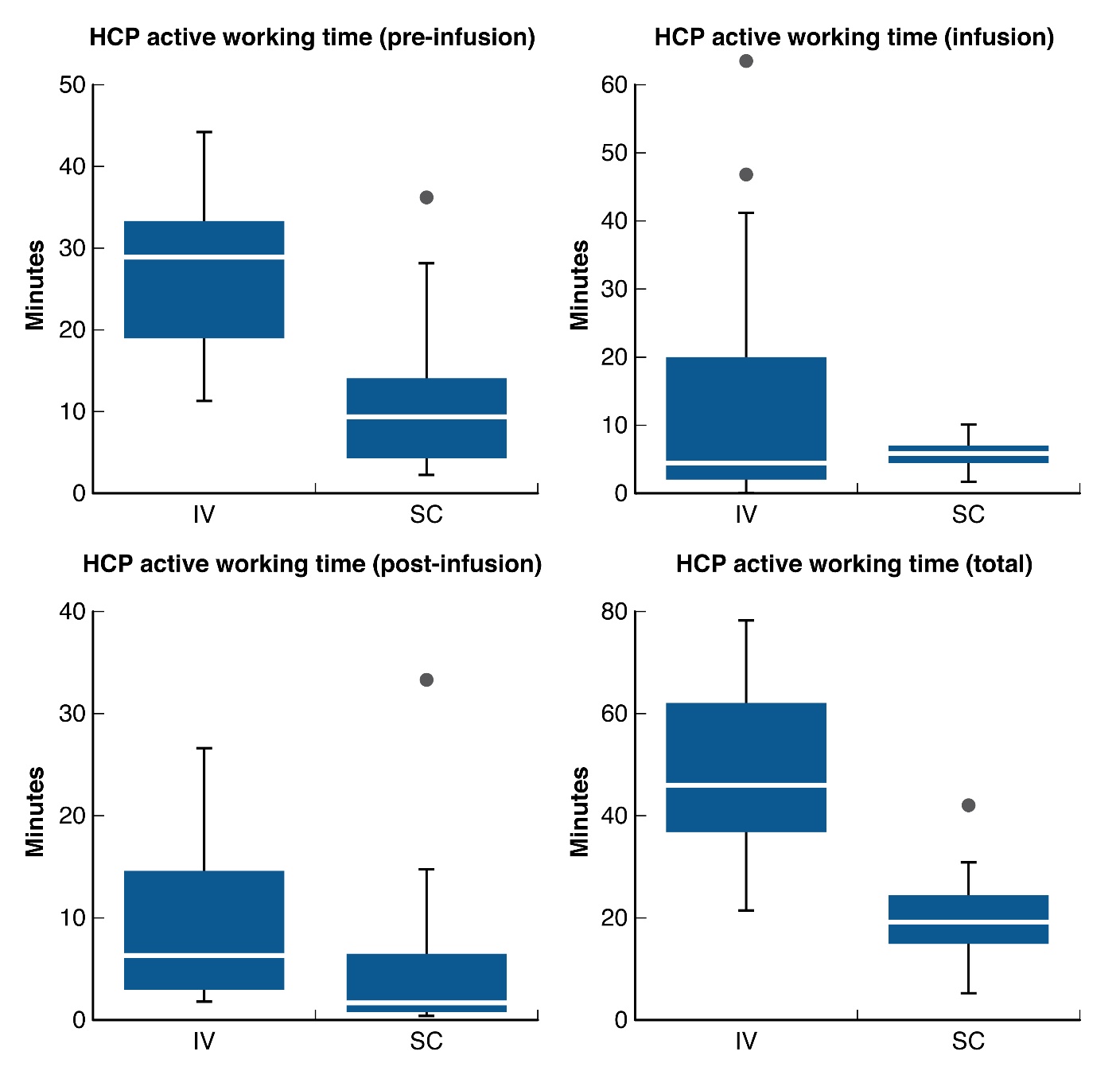


Supporting Figure S2. Box and whiskers plots relative to HCP active working time in each administration phase.

HCP = healthcare professionals; IV = intravenous; SC = subcutaneous


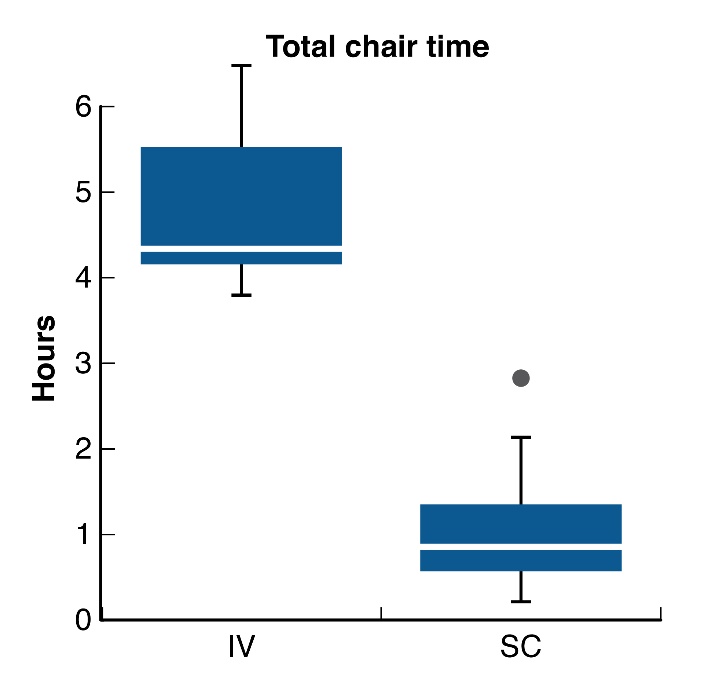


Supporting Figure S3. Box and whiskers plot relative to chair times.

IV = intravenous; SC = subcutaneous
